# Is a blunt sword pointless? Tooth wear impacts puncture performance in Tasmanian devil canines

**DOI:** 10.1242/jeb.246925

**Published:** 2024-01-31

**Authors:** Tahlia I. Pollock, David P. Hocking, Alistair R. Evans

**Affiliations:** ^1^ The Palaeobiology Research Group, School of Earth Sciences, University of Bristol, Bristol BS8 1QU, UK; ^2^ School of Biological Sciences, Monash University, Melbourne, VIC 3800, Australia; ^3^ Department of Zoology, Tasmanian Museum and Art Gallery, Hobart, TAS 7000, Australia; ^4^ Museums Victoria Research Institute, Museums Victoria, Melbourne, VIC 3001, Australia

**Keywords:** Tooth wear, Tooth morphology, Puncture performance, Mammal, Canine

## Abstract

As teeth wear, their shapes change and functional features can be dulled or lost, presumably making them less effective for feeding. However, we do not know the magnitude and effect of this wear. Using Tasmanian devil canines as a case study, we investigated the impact of wear on puncture in pointed teeth. We measured aspects of shape impacted by wear (tip sharpness, height and volume) in teeth of varying wear followed by 3D printing of real and theoretical forms to carry out physical puncture tests. Tooth wear acts in two ways: by blunting tooth tips, and decreasing height and volume, both of which impact performance. Sharper tips in unworn teeth decrease the force and energy required to puncture compared with blunter worn teeth, while taller unworn teeth provide the continuous energy necessary to propagate fracture relative to shorter worn teeth. These wear-modulated changes in shape necessitate more than twice the force to drive worn teeth into ductile food and decrease the likelihood of puncture success.

## Teaser

**Summary:** Teeth change shape and lose functional features as they wear; quantification of the magnitude and effect of this wear demonstrates that worn canine teeth require more than twice the force to drive into ductile food, and may not puncture the food.

## INTRODUCTION

Throughout an animal's lifetime, their teeth will experience countless interactions with food items and opposing teeth, causing them to wear. As teeth wear, their shape changes and functional features can be dulled or lost altogether (Van Valkenburgh, 1988; Evans, 2005; Evans and Sanson, 1998). Once-sharp blades may become ineffective at cutting through plant material or previously elongate pointed teeth may struggle to puncture prey. However, this has not been quantified experimentally and the magnitude and effect of this wear on tooth performance is not known.

Often the first point of contact between predator and prey, canine teeth are used to bite into a range of tissues. During feeding, canines may encounter one or a combination of grit-covered hides, flesh and bone, exoskeleton, hard-shelled prey and even fibrous plant materials, each causing wear (Van Valkenburgh, 1988; Ewer, 1973; Van Valkenburgh, 1996; Van Valkenburgh and Ruff, 1987; Christiansen and Wroe, 2007; Pollock et al., 2021c). As a canine tooth wears, the sharp tip (and sometimes edges) will blunt, tooth height and volume decrease, and robustness increases (Van Valkenburgh, 1988; Van Valkenburgh and Hertel, 1993). Previous research shows that tooth tip sharpness, volume and robustness impact puncture performance (Evans and Sanson, 1998; Crofts et al., 2019; Anderson, 2018; Freeman and Lemen, 2007; Freeman and Weins, 1997). As tools primarily used for puncture, canine teeth represent an excellent study system to investigate the relationship between tooth wear and performance. While the impact of tooth wear is expected to be especially important for older individuals, breakage and subsequent wear can happen to carnivores at all stages of life and in effect speed-up tooth wear (Van Valkenburgh et al., 2019). Because of their elongate form, canine teeth are the most frequently broken tooth in a carnivore's tooth row (Van Valkenburgh, 1988). Hence, understanding the ‘cost’ of a worn or broken canine is of particular importance.

Here, we quantified aspects of tooth shape impacted by wear in Tasmanian devil (*Sarcophilus harrisii*) (Boitard 1841) canines. Devils are an ideal candidate, as their diet and feeding behaviours expose their teeth to high wear, the degree and range of which are well documented (Andersen et al., 2017; Jones and Barmuta, 1998; Pemberton et al., 2008; Pemberton, 1990; Jones, 1995; Pollock et al., 2021a,b). To experimentally quantify the relationship between tooth wear and performance, we 3D printed a series of real and theoretical canine forms of varying wear states and subjected them to physical puncture tests.

## MATERIALS AND METHODS

### Quantifying tooth wear

We selected 38 Tasmanian devil canine teeth that spanned the range of tooth wear variation documented in the literature (Pemberton, 1990; Pollock et al., 2021b; Jones et al., 2008; Lachish et al., 2009). Teeth were sourced from multiple museum collections [Museums Victoria (NMV), the Tasmanian Museum and Art Gallery (TMAG) and the Australian Museum (AMS)]. Each tooth was coded according to a pre-existing tooth wear index and assigned to one of the five wear categories (Pollock et al., 2021b) (Table S1).

For digitisation, specimens were moulded and cast with silicone rubber impression material (Flexitime Correct Flow A-silicone, Heraeus, Hanau, Germany) and gypsum dental stone (Alpha-rock golden brown premium quality gypsum, AlphaBond Dental, Roseville, NSW, Australia). Casts were scanned on a Laser Design DS-Series 2025 3D scanner with an RPS-120 laser probe (620 nm) at a point spacing of 10 μm. Point clouds generated were merged into a single mesh in Geomagic Wrap 2015 (Geomagic, Santa Clara, CA, USA).

**Fig. 1.**
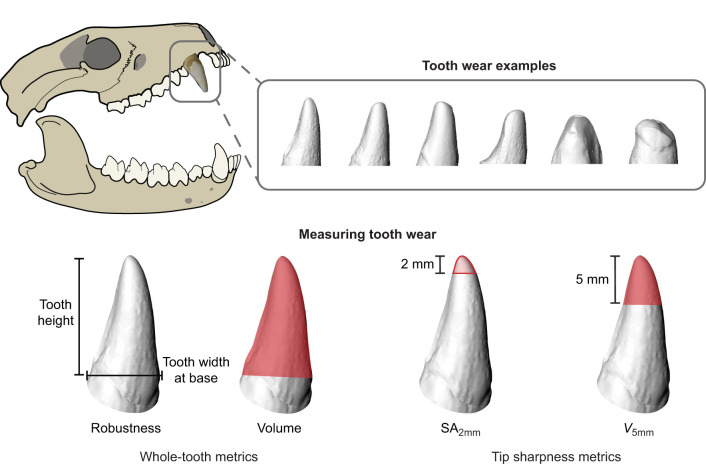
**Tooth wear examples in Tasmanian devil *Sarcophilus harrisii* and tooth shape metrics used to capture aspects of wear in this study.** Whole-tooth metrics: robustness (tooth height/tooth width at base) and tooth volume (mm^3^). Tip sharpness metrics: surface area (mm^2^) at 2 mm distance from tooth tip, SA_2mm_; and volume (mm^3^) at 5 mm distance from tooth tip, *V*_5mm_.

Tooth shape measurements were taken in Rhinoceros 5 (McNeel North America, Seattle, WA, USA). We measured tooth height from the tip of the tooth to the highest point of the boundary of the enamel, tooth width at the base of the tooth, and calculated tooth volume. We also quantified the surface area of the tip of the tooth at a distance of 2 mm from the tip (SA_2mm_) and the volume of the tip of the tooth at a distance of 5 mm from the tip (*V*_5mm_). By measuring tip surface area, we are quantifying the amount of a tooth's surface in contact with the substrate, which previous research on puncture mechanics has identified as important for force transmission and stress, which impact crack initiation (Evans and Sanson, 1998; Anderson, 2018; Lucas, 1982; Crofts et al., 2020). The smaller the surface area of tooth in contact with the substrate, the higher the stress for a given force and the greater the likelihood of fracture. Additionally, measuring tip volume (*V*_5mm_) and tooth volume enables us to quantify the amount of tooth being driven into the substrate and so the amount of substrate that needs to deform or be displaced, which has been highlighted as important for continued penetration (Evans and Sanson, 1998; Anderson, 2018). The less substrate that needs to be deformed or displaced, the fewer atomic bonds in the material need to break to continue penetration. We expected that our measures of tip sharpness (SA_2mm_ and *V*_5mm_) would be highly correlated; however, the inclusion of tip volume allowed us to examine the relationship between volume and puncture independent of tooth height in our controlled initial puncture experiments (described below), which is not the case for total tooth volume. Together, these metrics capture the aspects of tooth shape impacted by wear: tip sharpness (SA_2mm_ and *V*_5mm_), tooth height, volume and robustness (tooth height/tooth width) (Fig. 1). Focusing on a single tooth within a model species allowed us to experimentally control for size-related differences in our measures of tooth shape, and so we used absolute values. However, when comparing measures of tooth shape across taxonomically broad datasets with disparate body sizes, applying a scaling factor or using scale-independent measures of shape would be more appropriate, especially for sharpness (Pollock et al., 2021c; Crofts et al., 2019; Evans et al., 2005; Hocking et al., 2017).

### Model creation and 3D printing

Theoretical tooth models were created that span the variation in wear observed (Fig. 1; Fig. S1). Models were generated in Rhinoceros 5 by selecting a single unworn tooth (museum specimen NMV C3242) and artificially wearing it by removing tooth height at specific intervals (5%, 10%, 15%, 20%, 30%, 40% and 50% of the tooth height). To simulate the worn tip, we generated a surface from the equation *y*=0.5*x*^0.2^, taken from (Evans et al., 2021), which describes a blunt-tipped pointed tooth. This was scaled and trimmed to fit the cut tooth and then merged in Geomagic to create the final theoretical model. Tooth shape metrics were measured for all theoretical models.

All theoretical models (eight in total) and eight real models (generated from the casts of museum specimens) were chosen for 3D printing and puncture testing. For each theoretical tooth model, we selected one real tooth model which had the most similar tip sharpness value (SA_2mm_), resulting in tooth models representing wear states: W0, W5, W10, W15, W20, W30, W40 and W50. To enable secure mounting and a consistent run length (total displacement) for puncture tests, tooth models were designed to have a 18×30×12 mm base block and a total height of 30 mm. Tooth models were trimmed at their base so that the resulting edge was flat and perpendicular to the central axis of the tooth. However, as the height of each tooth varied, to achieve the desired total model height (30 mm), the base edge of each model was extended via the ‘extrude’ function in Geomagic. The amount extruded differed for each tooth and was calculated by adding the block height (12 mm) and tooth height (e.g. 12.35 mm for museum specimen NMV C2652) and subtracting it from the desired model height (30 mm). Once each tooth edge was extruded, the block was attached. One copy of each model was printed using a stereolithography (SLA) 3D printer (Form 3, Formlabs, Somerville, MA, USA) in Formlabs Standard Grey resin with a layer thickness of 0.050 mm. See Fig. S1 for model creation workflow.

### Puncture tests

To remove the inherent variability of using a biological substrate such as animal tissue, we used a gelatine analogue designed to mimic human skin and muscle tissue (Medical Gelatine #2, Humimic Medical, Greenville, SC, USA; material properties: density 923.468ρ kg m^−3^, speed of sound 1457.42 m s^−1^, Young's modulus 0.26 MPa, firmness 308 g and needle resistance 0.38 N, Humimic website). Gelatine was moulded and cast into 50×50×50 mm blocks for puncture tests.

A force tester (Instron 5982 Universal Testing Machine, Instron Mechanical Testing Systems, Norwood, MA, USA) with a S40A 50 kg load cell (HBM, Darmstadt, Germany) was used to measure the force (N) and displacement (mm) required by each tooth model to puncture the gelatine substrate. For each test, the tooth model was secured with a clamp attached to the load cell and the substrate placed on a platform below so that there was ∼1 mm between substrate and tooth tip. The set-up was designed so that the tooth moved downwards into the substrate at a constant speed of 2 mm s^−1^ for a distance of 18 mm, referred to as a ‘run’. Between each run, tooth models were cleaned, and the substrate replaced. For all tooth models, we performed six replicates of each run. The force and displacement output were recorded at 10 measurements per second by the data logger program EVIDAS Essential (v.1.3.0, HBM).

To assess puncture success, for each replicate, we photographed and visually inspected the gelatine block immediately after the run as well as 20 min after the run.

### Analysis

Force and displacement data for each replicate were processed and analysed in Excel (v.16.74, Microsoft) and using the RStudio statistical and graphical environment (R v.4.0.3, RStudio v.1.3.1093, http://www.R-project.org/). As previous research linked tip sharpness to the crack-initiation phase of puncture, and tool height and volume to the continued penetration phase, we separated our analyses into two parts (Evans and Sanson, 1998; Anderson, 2018; Lucas, 2004). First, to assess how tooth wear impacts initial contact/fracture between tooth and substrate, we calculated the maximum force (N) and energy (N mm; calculated as the area under the force–displacement curve) for each tooth model for the first 5 mm of the run (displacement 5 mm) and plotted these against measures of sharpness (SA_2mm_ and *V*_5mm_). Then, to assess how tooth wear impacts the complete bite/continued penetration, we calculated the maximum force and energy to a displacement equivalent to the tooth height of each model and plotted this against tooth height*,* tooth volume and tooth robustness (tooth height/tooth width), respectively. For each tooth model, maximum force and energy values for each replicate were averaged. Puncture was recorded as successful if there was visible damage to the cube, and unsuccessful if there was no visible damage (when substrate deformed around the tooth but did not break).

## RESULTS AND DISCUSSION

**Fig. 2.**
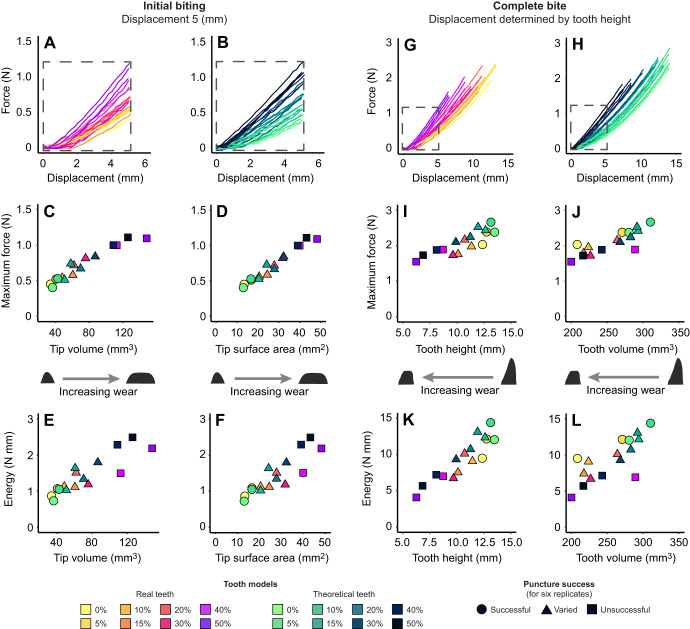
**Puncture performance results for initial biting (left) and complete bite analysis (right).** The force vs displacement traces for all replicates trimmed to the first 5 mm of the run (A,B) and for the full height of each tooth model (G,H). For penetration of the first 5 mm, maximum force (C,D) and energy (E,F) increase with wear metrics (tip volume and tip surface area). For complete tooth penetration, both maximum force (I,J) and energy (K,L) decrease with wear (tooth height and tooth volume). Data are for real tooth models (Real) and theoretical tooth models (Theo) at eight wear states (0% to 50%). Shapes denote combined puncture success for all replicates (successful, varied, unsuccessful).

We observed two patterns between tooth wear and puncture performance. For initial contact/fracture (displacement of 5 mm), as tooth wear increases (i.e. tips become blunter) so does the maximum force and energy (Fig. 2A–F), with significant positive correlations observed in real and theoretical tooth models for all sharpness metrics (*R*^2^>0.82, *P*<0.01; see Table S3 for full output of all statistical tests). For the SA_2mm_ sharpness metric, having a very worn tip compared with an unworn tip increased maximum puncture force by approximately 240%, where our highest wear theoretical (Theo 50%) and real (Real 50%) tooth models required 1.11 N and 1.09 N, respectively, and our unworn theoretical (Theo 0%) and real (Real 0%) tooth models required 0.40 N and 0.45 N, respectively. In theoretical models, for a 30.11 mm^2^ increase in bluntness (SA_2mm_) we found a 0.71 N increase in puncture force (Theo 0%, 13.10 mm^2^; and Theo 50%, 43.21 mm^2^) and for real models we found a 34.88 mm^2^ increase in bluntness increased force by 0.64 N (Real 0%, 13.34 mm^2^ and Real 50%, 48.22 mm^2^). A similar pattern was observed for sharpness metric *V*_5mm_ (see Table S2).

In contrast, for the complete bite/continued penetration (displacement equivalent to tooth height), as tooth wear increased (i.e. height and volume decreasing and robustness increasing), maximum force and energy decreased (Fig. 2G–L), with significant negative correlations observed for tooth height in real and theoretical tooth models (*R*^2^>0.70, *P*<0.01; see Table S3 for full output of all statistical tests) and also for tooth volume in theoretical (*R*^2^>0.95, *P*<0.01) but not real tooth models (*P*>0.05). Having a very worn tooth compared with an unworn tooth decreased the maximum puncture force by approximately 130%, where our highest wear theoretical (Theo 50%) and real (Real 50%) tooth models required 1.72 N and 1.54 N, respectively, and our unworn theoretical (Theo 0%) and real (Real 0%) tooth models required 2.37 N and 2.03 N, respectively. In theoretical models, for a 6.61 mm decrease in tooth height we found a 0.65 N decrease in puncture force (Theo 0%, 13.10 mm; and Theo 50%, 43.21 mm) and in real models, a 6.52 mm decrease in tooth height decreased puncture force by 0.83 N (Real 5%, 12.74 mm; and Real 50%, 6.22 mm; using Real 5% in this instance as it has the highest height value). See Table S2 and Fig. S2 for tooth volume and tooth robustness.

For both real and theoretical tooth models, as wear increased, the likelihood of puncture decreased (Fig. 2). Unworn and lightly worn tooth models (W0 and W5) were observed to successfully puncture the substrate in all replicates, while heavy wear models (W40 and W50) did not puncture in any replicates. Models with intermediate wear (W10–W30) displayed varying puncture success (Table S2).

Our results demonstrate experimentally the impact of tooth wear on puncture performance in pointed teeth. We show that wear acts two ways, by blunting tooth tips and decreasing the total tooth height and volume, all of which influence puncture performance.

During initial contact/fracture, increased wear and tip bluntness result in increased force and energy. For a given displacement (5 mm), the sharp tips of the less worn tooth models have a smaller surface area of contact with the substrate than the blunt tips of more worn teeth. This concentrates the force on a smaller surface area, resulting in increased stress in the substrate and likelihood of breaking the atomic bonds in the material (Anderson, 2018; Lucas, 1982; Crofts et al., 2020; Lucas and Luke, 1984). Previous work investigating relationships between shape and performance in pointed tooth forms have found the same general pattern between tip sharpness and puncture performance (Evans and Sanson, 1998; Crofts et al., 2019; Freeman and Lemen, 2007; Freeman and Weins, 1997; Freeman, 1992; Whitenack and Motta, 2010). However, we demonstrate it relative to tooth wear for the first time and are also able to show that, in Tasmania devil canine teeth, the approximately 3× increase in tip surface area (SA_2mm_) between an unworn tooth (W0) and a very worn tooth (W50) increases force approximately 2.5×.

We observed an inverse relationship between wear and puncture performance for the complete bite, where increased tooth wear (loss of tooth height and volume and increased robustness) decreased the force and energy needed. In ductile and tough materials like the medical gelatine used in this study and vertebrate skin and muscle, the applied force initially results in food deformation. Once initiated, a crack requires a significant amount of energy to propagate through the tough material (Anderson, 2018; Crofts et al., 2020; Lucas, 2004; Freeman and Lemen, 2006; Evans and Pineda-Munoz, 2018). As a tooth is pushed in, the material will continue to deform until the stress is sufficient to initiate a crack (initial fracture), and continued contact is necessary to propagate the crack (continued penetration) (Anderson, 2018; Lucas and Luke, 1984). In both these scenarios, if the tooth is not tall enough (or sharp enough), neither crack formation nor propagation will occur. Hence, we demonstrate that height is an important aspect of tooth shape related to puncture performance in animals that bite into ductile materials. Changes to canine tooth height, either from wear within a species or from morphological differences between species (e.g. giant panda versus a sabre-toothed felid like *Smilodon*), are likely to impact puncture performance.

These wear-modulated changes in tooth features (sharpness, height, volume and robustness) occur concurrently as a tooth wears throughout an animal's life; in combination, they make worn teeth less likely to successfully puncture prey. Put simply, there appears to be a minimum sharpness and tooth height required to initiate and propagate cracks in ductile and tough materials; worn teeth have neither, making them less effective. We see this borne out in the puncture success of our tooth models, where unworn and light wear models (W0 and W5) punctured in all replicates and very worn models (W40 and W50) did not puncture in any.

Tasmanian devils present a particularly interesting case study as, unusually for mammals, their canine teeth continue to erupt throughout their lives, and this over-eruption has been documented as a proxy for age (Pollock et al., 2021b; Jones et al., 2008; Lachish et al., 2007; Lazenby et al., 2018; Bell et al., 2020). Canine over-eruption in devils has been shown to maintain tooth height and may help compensate for their high wear diets (Jones, 2023) and is an interesting avenue for future research to explore.

This study quantified the magnitude of the effect of tooth wear, showing that worn teeth require more than twice the force to drive into ductile food, and in fact may not puncture the food. Therefore, there may be strong limits on feeding performance of individuals with worn teeth, reducing efficiency or requiring dietary change. Our findings have wide reaching implications for tooth performance in carnivores; for example, in older individuals, which are likely to have a higher degree of tooth wear, but also for carnivores at all stages of life which through tooth breakage and subsequent wear will be impacted (Van Valkenburgh, 1988; Van Valkenburgh et al., 2019).

## Supplementary Material

10.1242/jexbio.246925_sup1 Supplementary information

Table S1. Specimen information for all canine teeth measured for this study including tooth wear category (from Pollock *et al.* 2021), and parameters measured: tooth height (mm), tooth width (mm), tooth robustness (tooth height/tooth width), tip sharpness: surface area 2 mm from tip (mm^2^), tooth volume (mm^3^), and tip sharpness: volume 5 mm from tip (mm^3^).

Table S2. Tooth puncture performance values calculated for each replicate for all tooth models tested. Including Maximum force (N) and energy (Nmm), calculated from the full run (displacement 18 mm), run chopped to tooth height (displacement equivalent to tooth height (mm)), and run chopped to 5 mm (displacement equivalent to 5 mm)).

Table S3. Statistical output for all regressions undertaken in this study between performance metric(s) and aspects of tooth shape.

Table S4. Raw force trace output (displacement (mm) and force (N)) for each tooth model replicate.
