# Supplementary material for: Is a blunt sword pointless? Tooth wear impacts puncture performance in Tasmanian devil canines
Source: J Exp Biol. 2024 Jan 31;227(3):jeb246925. doi: 10.1242/jeb.246925 (PMC10917061; doi:10.1242/jeb.246925)
Supplement: Supplementary information [file jexbio-227-246925-s1.pdf]

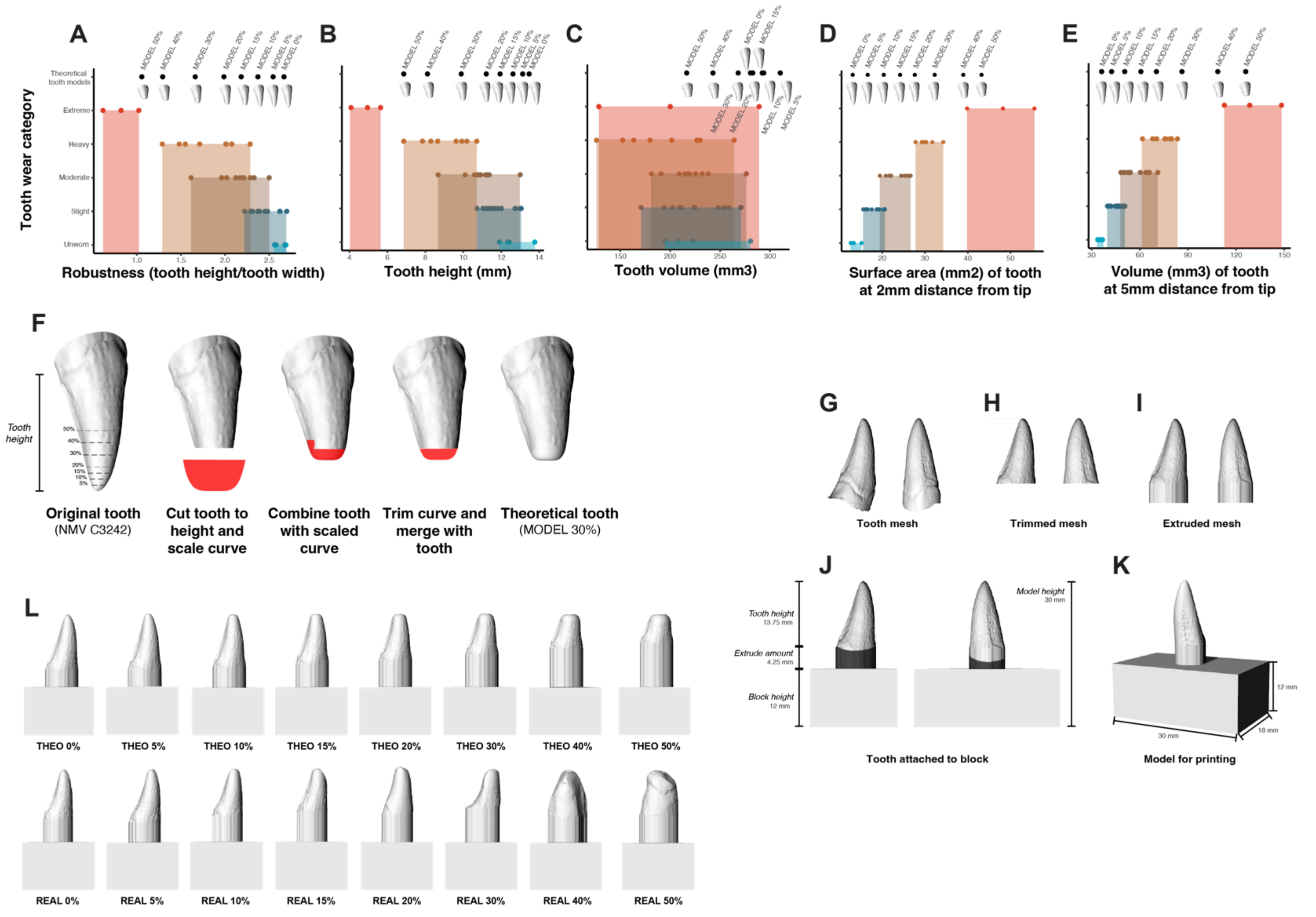

Fig. S1. Tooth wear categories and tooth shape metrics: (**A**) robustness, (**B**) tooth height, (**C**) tooth volume, (**D**) sharpness surface area, and (**E**) sharpness volume. (**F**) Theoretical tooth creation workflow shown on NMV C6242. Workflow for creating models for 3D printing as shown on tooth NMV C6242: (**G**) tooth mesh, (**H**) trimming, (**I**) extruding, (**J**) attaching to block, and (**K**) final model for printing.

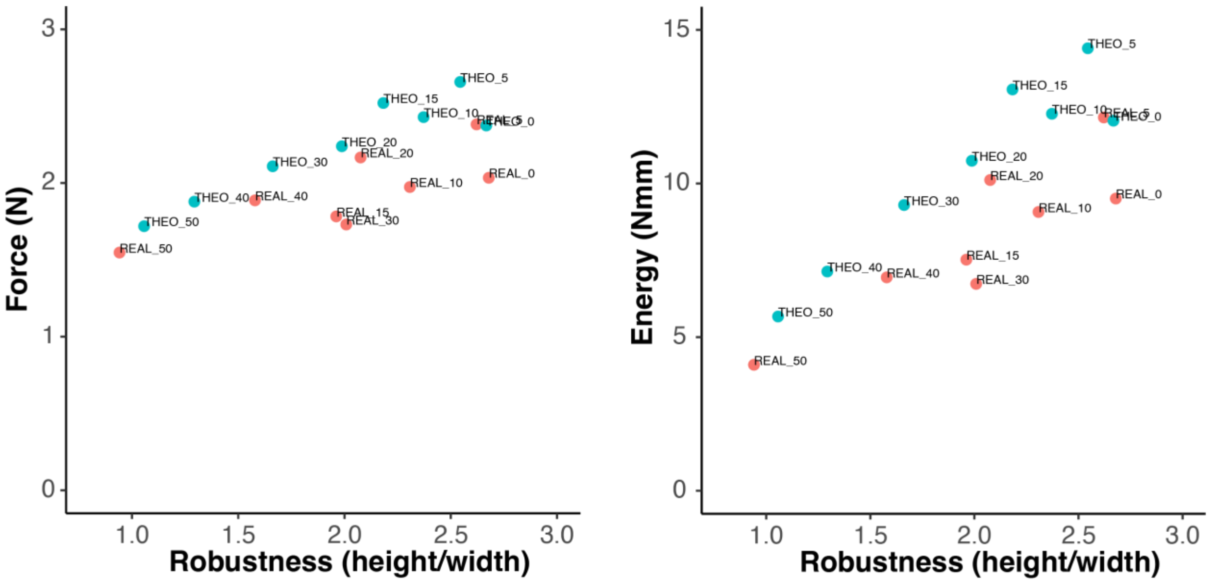

Fig. S2. Performance metrics (calculated from the whole bite) vs. robustness

Journal of Experimental Biology • Supplementary information

Table S1. Specimen information for all canine teeth measured for this study including tooth wear category (from Pollock *et al.* 2021), and parameters measured: tooth height (mm), tooth width (mm), tooth robustness (tooth height/tooth width), tip sharpness: surface area 2 mm from tip (mm^2), tooth volume (mm^3), and tip sharpness: volume 5 mm from tip (mm^3).

Available for download at
https://journals.biologists.com/jeb/article-lookup/doi/10.1242/jeb.246925#supplementary-data

Table S2. Tooth puncture performance values calculated for each replicate for all tooth models tested. Including Maximum force (N) and energy (Nmm), calculated from the full run (displacement 18 mm), run chopped to tooth height (displacement equivalent to tooth height (mm)), and run chopped to 5 mm (displacement equivalent to 5 mm)).

Available for download at
https://journals.biologists.com/jeb/article-lookup/doi/10.1242/jeb.246925#supplementary-data

Table S3. Statistical output for all regressions undertaken in this study between performance metric(s) and aspects of tooth shape.

Available for download at
https://journals.biologists.com/jeb/article-lookup/doi/10.1242/jeb.246925#supplementary-data

Table S4. Raw force trace output (displacement (mm) and force (N)) for each tooth model replicate.

Available for download at
https://journals.biologists.com/jeb/article-lookup/doi/10.1242/jeb.246925#supplementary-data

Journal of Experimental Biology • Supplementary information
